# Individual differences in the effect of menstrual cycle on basal ganglia inhibitory control

**DOI:** 10.1038/s41598-019-47426-8

**Published:** 2019-07-30

**Authors:** Esmeralda Hidalgo-Lopez, Belinda Pletzer

**Affiliations:** Department of Psychology and Centre for Cognitive Neuroscience, University of Salzburg, Hellbrunnerstr. 34, 5020 Salzburg, Austria

**Keywords:** Neuroscience, Cognitive control

## Abstract

Basal ganglia (BG) are involved in inhibitory control (IC) and known to change in structure and activation along the menstrual cycle. Therefore, we investigated BG activation and connectivity patterns related to IC during different cycle phases. Thirty-six naturally cycling women were scanned three times performing a Stop Signal Task and hormonal levels analysed from saliva samples. We found an impaired Stop signal reaction time (SSRT) during pre-ovulatory compared to menses the higher the baseline IC of women. Blood oxygen level dependent (BOLD)-response in bilateral putamen significantly decreased during the luteal phase. Connectivity strength from the left putamen displayed an interactive effect of cycle and IC. During pre-ovulatory the connectivity with anterior cingulate cortex and left inferior parietal lobe was significantly stronger the higher the IC, and during luteal with left supplementary motor area. Right putamen’s activation and left hemisphere’s connectivity predicted the SSRT across participants. Therefore, we propose a compensatory mechanism for the hormonal changes across the menstrual cycle based on a lateralized pattern.

## Introduction

Inhibitory control (IC) is defined as the ability to suppress active cognitive processes for the purpose of selecting one action over another, including actions that have already been initiated^1–3^. It has been suggested to rely on a prefrontal cortex-basal ganglia (PFC-BG) network^4–7^. Although most studies focus on the frontal substrates (ventro medial prefrontal cortex-VMPFC-, including the inferior frontal gyrus –IFG-, and the supplementary motor area –SMA-), the BG have also been shown to be responsible for IC^4,8,9^. Among the implicated subcortical structures the sub thalamic nucleus (STN)^4,5,10,11^ and the striatum^12,13^ are the most studied. As primary input station to the BG, the striatum plays a key role in IC^14,15^ and increased activation during successfully inhibited trials has been observed^4,8,9,16^. Likewise, IC is impaired when BG is lesioned^17^, or dysfunctional, related to several neurological disorders^18–20^.

Female sex hormones are known to affect multiple neurotransmitter systems, such as dopamine, serotonin or gamma-Aminobutyric acid (GABA), and modulate executive functions across the menstrual cycle^21–24^. Accordingly, there is some evidence that in women, IC and the associated brain activation and connectivity changes across the menstrual cycle. However, research in this area is scarce and the results are inconsistent. Notably, some of the evidence does not come from studies on IC per se, but from studies on impulsivity, since low IC has been consistently related to increased impulsive behavior^25^. A variety of behavioral paradigms have been used to measure IC. One of them is the Stop Signal Task (SST), in which participants need to withhold an already started response. The time to stop an already initiated response is referred to as Stop signal reaction time (SSRT)^26–28^. This estimate of the covert inhibitory process provides a behavioral index of the individual IC ability, with longer SSRTs indicating lower IC. Behaviorally, an impaired performance in the SST, has been observed during the pre-ovulatory phase correlated to the increased estradiol levels that characterize this phase, compared to menses (when both estradiol and progesterone are low) and luteal phase (when progesterone levels are the highest and estradiol maintains medium levels)^29^. However, other studies report a decrease in impulsive behavior during the pre-ovulatory phase compared to menses in some women^30^. Moreover, the role of progesterone in the luteal phase with regards to IC is widely unexplored. Some studies have reported an increase in impulsivity during the luteal phase or just before menses^31^, while others report a decrease during the luteal phase in some women^32^.

Regarding neuroimaging studies, in go/no go paradigms, brain activity has been reported to increase during IC in left precentral gyrus and left precuneus^33^, medial orbitofrontal cortex (OFC) and decrease in lateral OFC^34^ and IFG^35^ during pre-ovulatory compared to luteal phase; and decrease in anterior cingulate cortex (ACC) during luteal compared to menses and pre-ovulatory phases^36^. Furthermore, reduced functional connectivity was found between left hemispheric frontal areas as well as frontoparietal interhemispheric connectivity during the luteal phase associated to high levels of both estradiol and progesterone^36^. Although variations in brain activity during IC across the menstrual cycle are usually reported in frontal areas^34,35,37^ the BG are known to change in structure, activation and connectivity across different cycle phases^23,38,39^. Additionally, previous studies have shown an increased activation in women’s BG compared to men’s during IC processes^40,41^. However, despite the cumulative evidence pointing at the BG as a suitable candidate to study, up until now no research has focused on the cycle dependent changes in this area during IC. Thus, we are the first to explore the BG as region of interest (ROI) in order to investigate the influence of female sex steroids on the IC brain network.

Moreover, evidence suggests there is a differential effect of sex hormone levels depending on the individual level of impulsivity. During a reward paradigm study, Diekhof *et al*.^30^ showed an estradiol dependent decrease in impulse behavior during the pre-ovulatory phase compared to menses that was specific for women with lower trait of impulsiveness. A more recent study by Roberts *et al*.^32^ also found a differential effect on attention deficit hyperactivity disorder (ADHD) symptoms across the menstrual cycle depending on women’s level of impulsiveness. Specifically, impulsive behaviour was higher particularly for those women with high trait impulsivity, during menses and post-ovulatory compared to mid luteal. They found this effect related to the combination of lower estradiol levels and higher progesterone levels. In line with these between subjects differences related to the baseline level of IC, a number of studies have shown an increased SSRT in clinical disorders that imply an impaired IC and altered BG, such as obsessive compulsive disorder (OCD), ADHD or substance abuse disorders (for a review, see 7). Accordingly, differences in brain activity and connectivity patterns have been described for subjects with different levels of IC^42^ and previous studies found different activation of the BG (among other structures) between subjects with short vs. long SSRT^43,44^. It is essential to outline these different patterns and its functional substrates in women in order to identify risk factors for mentioned disorders^7^.

Although the previous referenced studies evidences the importance of taking into account both intra and inter individual factors that modulate the IC (baseline IC and cycle phase respectively, in this case), no research has so far addressed, whether menstrual cycle dependent changes in IC depend on women’s baseline IC. If so, this may account for inconsistencies between studies. Therefore, our aim is to examine how BG activity and connectivity mediate IC throughout the menstrual cycle, also taking into account individual IC, measured by the baseline SSRT. We hypothesize an altered SSRT associated to altered BG activation and connectivity with the IC network during the pre-ovulatory phase of the menstrual cycle. We expect these effects to be opposite in women with different baseline IC.

## Results

### Cycle phase and hormone levels

In order to compare sex hormone levels between the different cycle phases, the fixed factor cycle phase was modelled for dependent variables estradiol and progesterone, respectively in the context of a linear mixed effects model.

Estradiol was significantly higher in the pre-ovulatory phase compared to menses phase (*b* = 0.25, *SE*_b_ = 0.09, *t*_(34)_ = 2.92, *p* < 0.01) and in luteal phase compared to menses phase (*b* = 0.27, *SE*_b_ = 0.09, *t*_(34)_ = 3.04, *p* < 0.01); whereas it did not differ significantly between pre-ovulatory and luteal phases (*b* = 0.03, *SE*_b_ = 0.11, *t*_(35)_ = 0.29, *p* > 0.05). Progesterone was significantly higher in luteal phase compared to the menses phase (*b* = 0.55, *SE*_b_ = 0.09, *t*_(35)_ = 6.24, *p* < 0.001), and to the pre-ovulatory phase (*b* = 0.48, *SE*_b_ = 0.08, *t*_(35)_ = 5.80, *p* < 0.001); whereas it did not differ significantly between the pre-ovulatory phase and menses phase (*b* = 0.17, *SE*_b_ = 0.09, *t*_(35)_ = 1.98, *p* > 0.05) (all means are displayed in Table 1).

**Table 1:** Mean levels of salivary estradiol and progesterone in each cycle phase. N = 36. *M* = Mean; *SD* = Standard deviation.

	Estradiol (pg/ml)		Progesterone (pg/ml)	
	*M*	*SD*	*M*	*SD*
Menses	0.64	0.29	64.29	38.83
Pre-ovulatory	0.79	0.32	81.68	59.70
Luteal	0.81	0.34	205.79	149.97

### Behavioral performance

Table 2 presents behavioral performance indices across the different cycle phases of the menstrual cycle. As expected, since inhibited Stop and hit Go trials are assumed to be two competing independent processes, the Stop signal reaction time (SSRT) did not correlate with the Go reaction time (RT) (*cor* = −0.022, *t*_(106)_ = −0.23, *p* > 0.05). On the other hand, SSRT correlated with the mean Stop signal delay (SSD) (*cor* = −0.313, *t*_(106)_ = −3.40, *p* < 0.001), providing further evidence for the adequacy of the task tracking algorithm. Neither Go RT nor SSD were affected by the baseline inhibitory control (IC), cycle phase or its interaction (all |*b*| < 0.35, all *SE*_b_ < 0.18, all |*t*| < 1.85, all *p* > 0.05).

**Table 2:** Behavioral indices across the different cycle phases of the menstrual cycle. N = 36. RT: reaction time; SSD: Stop signal delay; SSRT: Stop signal reaction time; *M* = Mean; *SD* = Standard deviation.

	Go RT (ms)		Hit Stop (%)		SSD (ms)		SSRT (ms)	
	*M*	*SD*	*M*	*SD*	*M*	*SD*	*M*	*SD*
Menses	563.13	89.00	51.27	5.01	238.97	85.46	309.59	24.53
Pre-ovulatory	561.08	87.58	50.31	4.41	237.89	87.69	315.04	21.64
Luteal	572.92	96.36	51.36	5.25	251.05	90.36	306.22	36.55

**Figure 1:**
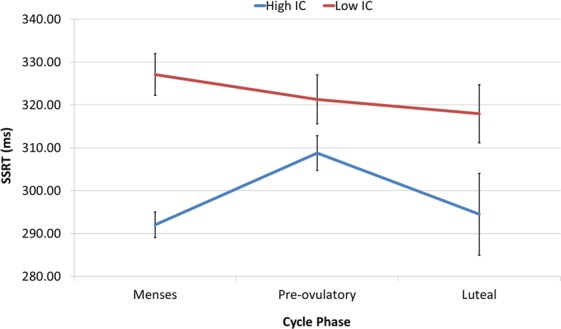
Interactive effect between cycle phase and baseline inhibitory control (IC) on the Stop signal reaction time (SSRT): Women that are faster during their menses, become slower during their pre-ovulatory phase, while women that are slower during their menses, become faster during their pre-ovulatory phase. Baseline IC as a covariate of interest was used as a continuous variable, but the sample is split by the median for displaying purposes. Error bars represent standard errors.

There was no main effect of cycle phase on SSRT (all |*b*| < 0.30, all SE_b_ < 0.20, all |*t*| < 1.50, all *p* > 0.05). However, we found an interactive effect between cycle phase (pre-ovulatory compared to menses) and baseline IC on the SSRT (*b* = −0.49, *SE*_b_ = 0.18, *t*_(67)_ = −2.75, *p* < 0.01). Women that are faster during their menses, become slower during their pre-ovulatory phase, while women that are slower during their menses, become faster during their pre-ovulatory phase (Fig. 1). Baseline IC did not affect menstrual cycle dependent changes during the luteal phase (all |*b*| < 0.35, all SE_b_ < 0.20, all |*t*| < 1.90, all *p* > 0.05) and none of the significant effects were related to hormone levels (all |*b*| < 0.20, all SE_b_ < 0.15, all |*t*| < 1.50, all *p* > 0.05).

### Neuroimaging results

#### Overall activation inhibited vs. non inhibited Stop trials

**Figure 2:**
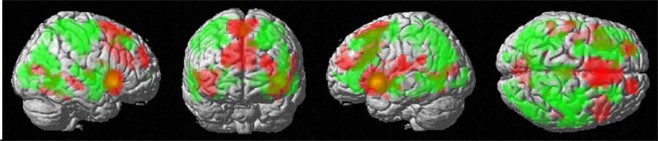
Overall activation during the Stop Signal Task: BOLD-response for successfully inhibited Stop trials vs. non-inhibited Stop trials in green. BOLD-response for non- inhibited Stop trials vs. successfully Stop trials in red. Uncorrected p < 0.001, pFWE < 0.05 at cluster level, and extent threshold of k = 40 voxels.

Compared to the non-inhibited Stop trials, the inhibited Stop trials strongly activated a large fronto-temporal and subcortical network including bilateral basal ganglia (BG) and hippocampus. Peaks of activation above threshold were located in the bilateral putamen and superior frontal gyri, left inferior frontal gyrus, right poscentral and left paracentral gyri, left parahippocampal area and bilateral temporal and occipital areas (Fig. 2, in green.)

Compared to the inhibited Stop trials, the non-inhibited Stop trials strongly activated a temporo-medial network including anterior cingulate cortex (ACC), bilateral insula, right middle temporal gyrus and subcortical structures (Fig. 2, in red.)

#### Region of interest (ROI) analysis: bilateral putamen

**Figure 3:**
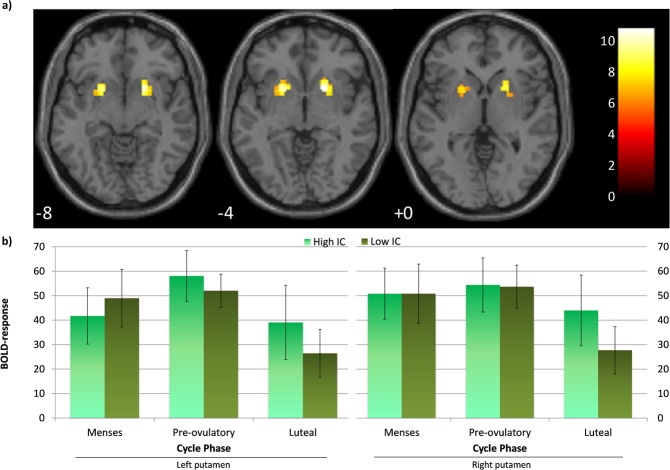
(**a**) Seed region for region of interest (ROI) and psychophysiological interaction (PPI) analysis: Bilateral putamen, peak coordinates [−18, 8, −5], [21, 11, −5]. (**b**) BOLD-response in the left and right putamen across the menstrual cycle: the BOLD-response significantly decreased during the luteal phase compared to the pre-ovulatory phase, irrespective of the baseline inhibitory control (IC). Baseline IC as a covariate of interest was used as a continuous variable, but the sample is split by the median for displaying purposes. Error bars represent standard errors.

For both left and right putamen we found a significant decrease in the blood oxygen level dependent (BOLD)-response during the luteal phase compared to the pre-ovulatory phase (*b* = −0.47, *SE*_b_ = 0.18, *t*_(33)_ = −2.69, *p* < 0.05 for the left and *b* = −0.38, *SE*_b_ = 0.18, *t*_(33)_ = −2.12, *p* < 0.05, for the right putamen; Fig. 3b). We did not observe any differences between pre-ovulatory phase and luteal phase vs. menses (all |b| < 0.32, all *SE*_b_ < 0.20, all |*t*| < 1.70, all *p* > 0.05). The main effects of baseline IC and its interaction with cycle phase were no significant for either left or right putamen BOLD-response (all |*b*| < 0.10, all *SE*_b_ < 0.20, all |*t*| < 0.50, all *p* > 0.05). None of the significant changes in activation across the menstrual cycle were related to hormone levels (all |*b*| < 0.05, all *SE*_b_ < 0.10, all |*t*| < 0.50, all *p* > 0. 05). The SSRT could be predicted by the right putamen BOLD-response (*b* = −0.21, *SE*_b_ = 0.09, *t*_(71)_ = −2.21, *p* < 0.05), but not by the left putamen (*b* = −0.14, *SE*_b_ = 0.09, *t*_(71)_ = −1.52, *p* > 0.05). The higher the activation of the right putamen, the shorter the SSRT, and therefore, the better performance.

#### Psychophysiological Interaction (PPI) analysis: bilateral putamen

**Figure 4:**
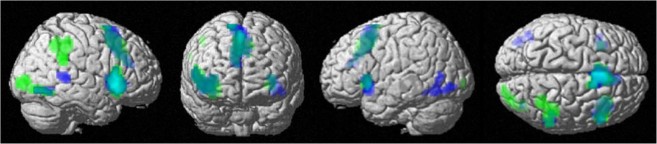
Overall connectivity with bilateral putamen irrespective of cycle phase and Stop signal reaction time (SSRT) for inhibited Stop trials: Overall connectivity network with left putamen [−18, 8,−5] in green, and with right putamen [21, 11, −5] in dark blue. Overlap areas in light blue. Uncorrected p < 0.001, pFWE < 0.05 at cluster level, and extent threshold of k = 40 voxels.

Overall connectivity patterns inhibited Stop vs. Non inhibited Stop: For the successfully inhibited Stop trials, the left putamen [−18, 8, −5] displayed significant connectivity with the ipsi- and contralateral insula, cingulate cortex, bilateral supplementary motor area (SMA), and contralateral supramarginal and calcarine gyri (Fig. 4, in green). The right putamen [21, 11, −5] showed a very similar network, including ipsi and contralateral insula, cingulate cortex, ipsilateral SMA and middle temporal area and bilateral fusiform gyri (Fig. 4, in dark blue). Connectivity from both putamens overlap in the ACC, SMA and bilateral insula (Fig. 4, in light blue).

Cycle phase effects and interactive effects with SSRT: We did not find any significant changes across menstrual cycle in the connectivity from left and right putamen for the non-inhibited vs. the inhibited Stop trials, or interactive effects between cycle phase and the baseline IC for the right putamen connectivity.

**Figure 5:**
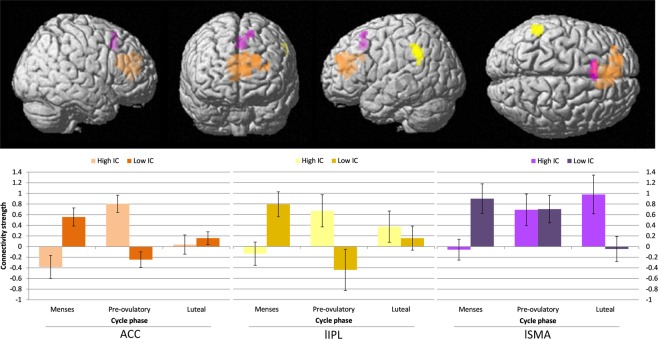
Interactive effect of cycle phase and baseline baseline inhibitory control (IC) on connectivity strength from the left putamen during inhibited responses compared to non-inhibited: During pre-ovulatory phase compared to menses connectivity strength between the left putamen and anterior cingulate cortex (ACC, in orange) and left inferior parietal lobe (lIPL, in yellow) was significantly higher for women with higher baseline IC, while it was decreased for women with lower baseline IC. During luteal phase compared to menses connectivity strength between the left putamen and left supplementary motor area (lSMA, in purple) was significantly higher for women with higher baseline IC, while it was decreased for women with lower baseline IC. Uncorrected p < 0.001, pFWE < 0.05 at cluster level, and extent threshold of k = 40 voxels. Baseline IC as a covariate of interest was used as a continuous variable, but the sample is split by the median for displaying purposes. Error bars represent standard errors.

Furthermore, when considering the baseline IC as a covariate, we found an interactive effect between cycle phase and baseline IC for connectivity of the left putamen to the ACC ([−6, 41, 16], *k*_E_ = 318, *T* = 5.67, *p*_FWE_ = 0.000; Fig. 5, in orange), left inferior parietal lobe (IPL) ([−54, −46, 37], *k*_E_ = 57, *T* = 5.51, *p*_FWE_ = 0.012; Fig. 5, in yellow) and left SMA ([−3, 20, 43], *k*_E_ = 40, *T* = 4.28, *p*_FWE_ = 0.047; Fig. 5, in purple). From menses to pre-ovulatory phase the connectivity between left putamen and ACC as well as between left putamen and left IPL decreased for women with lower IC, while it increased for women with higher IC. These effects in connectivity were not related to hormonal levels (all |*b*| < 0.20, all *SE*_b_ < 0.10, all |*t*| < 1.94, all *p* > 0.05). From menses to luteal phase the connectivity between left putamen and the left SMA decreased for women with lower IC, while it increased for women with higher IC. This interactive effect was not related to estradiol levels (*b* = 0.05, *SE*_b_ = 0.13, *t*_(68)_ = 0.34, *p* > 0.05), but to progesterone levels (*b* = −0.28, *SE*_b_ = 0.11, *t*_(69)_ = −2.58, *p* < 0.05). For women with lower IC, higher levels of progesterone related to weaker or negative connectivity, while for women with higher IC, higher levels of progesterone related to stronger connectivity.

Finally, the SSRT could be predicted by the connectivity of the left putamen to all of the former areas (*b* = 0.20, *SE*_b_ = 0.08, *t*_(71)_ = 2.57, *p* < 0.05 for the ACC; *b* = 0.19, *SE*_b_ = 0.08, *t*_(71)_ = 2.31, *p* < 0.05 for the IPL and *b* = 0.18, *SE*_b_ = 0.08, *t*_(71)_ = 2.16, *p* < 0.05 for the SMA). The stronger the connectivity between the left putamen and the ipsilateral ACC, IPL and SMA respectively, the longer the SSRT, hence the worse the performance.

## Discussion

The present study explored the interactive effect of cycle phase and baseline inhibitory control (IC) on women’s basal ganglia (BG) activation and connectivity during a Stop Signal Task (SST). Overall, a lateralized pattern emerged in putamens’ activation and connectivity, as a compensatory mechanism for hormonal changes across the menstrual cycle. Furthermore, depending on women’s baseline IC, sex steroids modulation showed opposite effects on behavior and connectivity.

Specifically, in behaviour, we observed an impaired Stop signal reaction time (SSRT) during the pre-ovulatory phase compared to menses, but only for those women who had better baseline IC. In a previous study^45^, we found a very similar pattern to this effect in the reaction time (RT) across cycle phases for the IC aspect of working memory. These results are in line with Roberts *et al*.^32^, who found higher impulsivity in women during menses and post-ovulatory compared to pre-ovulatory, but only for those with lower IC. They interpreted these results in relation to Jacobs & D’Esposito^46^, in which women with lower baseline levels of dopamine (and genotype risk for elevated trait impulsivity) showed better executive function during pre-ovulatory whereas those with higher baseline dopamine levels performed worse during this phase. It’s been shown before that the increase in dopamine related to higher estradiol levels during the pre-ovulatory phase could be responsible for the IC impairment, if it exceeds the optimal neurotransmitter levels for this specific process^29^. The differential effect of the baseline IC that we found could account for the inconsistencies in previous studies.

Consistent with previous findings, extensive activation during successful Stop trials compared to unsuccessful trials was observed in cortical and subcortical areas, including frontal areas and bilateral BG^5,41,47^. Remarkably, our global peaks of activation during successfully Stop trials we located within the BG. The previous neuroimaging literature on SST was mainly based on samples composed by men, but it has been shown before that women engage a different network during response inhibition. Related to a stronger top-down cognitive control strategy, an increased superior prefrontal and BG activation emerges in women compared to men, as well as a more bilateralized activation pattern, suggesting a greater role of the left hemisphere^40,41,48^.

Within the BG, we identified the bilateral anterior putamen as region of interest (ROI) based on the One-sample t-test. This area has already been shown to be of particular importance in IC and more active during the successfully inhibited responses^4,14,47^, especially in women^9,41,49^. Irrespective of the baseline IC, we found a significant decrease in the blood oxygen level dependent (BOLD)-response for the bilateral putamen during the luteal phase compared to the pre-ovulatory phase. Furthermore, the activation of the right putamen predicted the SSRT across participants. The higher the activation of the putamen, the shorter the SSRT and thus the better performance, as it has been already observed in previous studies^50^. Given that we observed the worst performance during pre-ovulatory phase, the increase in putamen activation during that phase may reflect a compensatory mechanism. In order to counteract detrimental effects of estradiol on performance, participants increased their putamen activation. Taking the higher IC women for example, in those who managed to compensate during the pre-ovulatory phase, the BOLD-response was higher, and the SSRT was preserved. However, for those women who could not compensate by increasing the putamen activation, the SSRT became longer during the pre-ovulatory phase compared to menses.

Since neither estradiol nor progesterone levels were related to these effects, it is possible that the putamen is compensating for the sex hormones modulation in other brain areas not included in the present study. Apart from the BG, multiple areas such as hippocampus, prefrontal cortex and primary sensory-motor cortex express estradiol and progesterone receptors, and are affected structurally and functionally by sex hormones (for a review, see 51). Moreover, the strong decrease in the BOLD-signal during the luteal phase suggests that progesterone is not only counteracting the estradiol effect, but over-balancing it (as suggested by Roberts *et al*.^32^), and therefore preventing the compensatory effect of the putamen, since it is no longer needed.

Regarding the connectivity for the bilateral putamen, it should be noted that the connectivity networks from both putamens’ overlap in those areas that have been reported before to be more active during the unsuccessful inhibited responses: anterior cingulate cortex (ACC), supplementary motor area (SMA) and bilateral insula^43,47^. Li *et al*.^48^ observed that some of the gender differences, specifically in ACC and pre-SMA, were due to an increased BOLD-response during error processing in unsuccessful compared to successful Stop responses for women. We did not find a main effect of the baseline IC or cycle phase on the bilateral putamens’ connectivity pattern when considered separately. However, the connectivity of the left putamen with the ACC, ipsilateral inferior parietal lobe (IPL) and SMA was modulated by cycle phase depending on the baseline IC. Likewise, we found these areas to be more active during non-inhibited trials compared to successfully inhibited trials. As suggested by Hirose *et al*.^52^ for a Go/No-go task, the left hemisphere could be playing a supplementary role for the efficiency of the task. Furthermore, the differences in connectivity strength between each of these areas predicted performance in the SST, with longer SSRT and therefore worse performance, the stronger the connectivity.

Both ACC and IPL have been observed to be connected to the putamen through structural, resting state and task based functional connectivity^53–56^. Interestingly, they both belong to the default mode network (DMN)^57^ and in the present study were “deactive” during the successfully inhibited trials. These findings are consistent with the suggestion that the ability to detach from or supress the DMN during a task is associated with better performance^58,59^, while the persistence of these areas’ activity results in a longer RT^60^ and precede Stop signal error^61^. Furthermore, Tian *et al*.^62^ observed and increased local synchronization (regional homogeneity) of resting state signals within the DMN including left IPL and related to longer SSRT. Given that the stronger connectivity predicted a longer SSRT, the impairment showed in behaviour the higher baseline IC during pre-ovulatory compared to menses relates to the increased connectivity strength between the left putamen and ACC/ipsilateral IPL, reflecting a failure to disconnect from the DMN. It would be interesting for further studies to explore whether changes of the DMN during resting state across the menstrual cycle also express a differential pattern depending on the baseline IC.

Consistent with data from previous studies the anterior putamen mediates the frontostriatal loops through the SMA among other structures, and both areas showed coupled activation during successful versus unsuccessful inhibition^14,16^. In contrast to the ACC and IPL, the SMA is known to participate in motor planning^7,50^. Accordingly, the increase in connectivity strength for high baseline IC women during pre-ovulatory phase compared to menses could point out a failure in breaking an initiated response, and therefore a delay in cancelling the motor action. This again may contribute to their worsened performance during that phase. However, the significant interactive effects in the connectivity with this area were found when comparing menses to luteal phase, which is similar to the putamens’ activation pattern. During the luteal phase, the SMA connectivity does not mirror behavioral performance. This may be explained as a response to the putamens’ drop in activation during this cycle phase. Moreover, this effect in connectivity was the only one related to hormonal values. Higher levels of progesterone were related to stronger connectivity for higher baseline IC women, whereas higher levels of progesterone were related to weaker connectivity for lower baseline IC women.

Although progesterone effects are less studied than estradiol, it is known to enhance gamma-Aminobutyric acid (GABA)_A_ receptor activity, increasing the effects of GABA. Furthermore it increases monoamine oxidase activity, thus decreasing monoamine levels, like norepinephrine, serotonin and dopamine (see 21 for a review). For some of these neurotransmitters a different baseline balance has been observed depending on participants’ trait impulsivity^63^. Likewise, other inter-individual factors like personality dimensions or abilities are accompanied by differences in both brain function and structure, including the variation in the baseline IC or impulsivity^7,64^. Inconsistencies in menstrual cycle research have been linked to different dopamine baseline levels^46,65^ and trait impulsivity has been related to striatal dopamine release in previous studies^63,66,67^. Therefore, these baseline differences between subjects with higher vs. lower IC may lead to opposite patterns of sex steroid modulation regarding the connectivity strength between crucial areas involved in IC.

In summary, our data suggest that sex steroids have opposite effects on behavior and connectivity depending on individual differences in IC. We observed a lateralized response in which the right putamen increases its activation, while the left putamen changes its connectivity to areas involved in the IC process in order to compensate hormonal effects. To the best of our knowledge this study is the first to explore the IC network along the menstrual cycle, focusing on the BG as ROI, and taking into account the inter-variability in IC among women.

## Materials and Methods

### Participants

Forty-four healthy right-handed women aged between 18 and 35 years old were recruited through social media and from the University of Salzburg as part of a larger study. Of those, 1 participant was excluded due to inconsistencies in the follow up report of her next cycle, 3 because of inconsistencies between hormone values and cycle phase as calculated based on self-reports and 1 due to bad quality of the functional magnetic resonance imaging (fMRI) preprocessing procedure. Behavioral data from 39 women was analyzed for the Stop Signal Task (SST) and from those 3 participants were excluded for poor task performance (See statistical analysis section for performance criteria). Accordingly, 36 women (*M*_age_ = 23.44, *SD* = 3.83) were included in the final analysis. All of them had regular menstrual cycle (*M*_cycle length_ = 28.31 days, *SD* = 2.22), defined as ranging between 21 and 35 days and less than 7 days of variability between individual cycle length^68^. Other exclusion criteria were neurological, psychiatric or endocrine disorders, having used hormonal contraceptives within the previous six months and being under medication treatment. All participants received either course credits or 30 € for their participation.

### Ethics statement

Experiments were approved by the University of Salzburg’s ethics committee and were conducted in accordance with the Code of Ethics of the World Medical Association (Declaration of Helsinki), and all participants gave their informed written consent to participate in the study. Upon arrival at the lab, participants were assigned a subject ID (VP001, VP002, etc.), which was used throughout the study.

### Procedure

Women were scanned three times across their menstrual cycle: (i) during menses (low progesterone and estradiol), (ii) in the pre-ovulatory phase (low progesterone and peak of estradiol levels), and (iii) during the mid-luteal phase (high progesterone and estradiol levels), order counter-balanced. The appointments were scheduled according to each participants’ cycle length and self-reports, and cycle phases were confirmed by follow up reports of the next cycle and ovulation tests (Pregnafix® Ovulationstest), which indicate a rise in luteinizing hormone before ovulation. Menses phase spanned from the second day of menstruation to seven days before ovulation (*M*_day_ = 4.00, *SD* = 1.48), pre-ovulatory appointments took placed when ovulation test showed a positive result on the day of testing or the day before (*M*_day_ = 12.36, *SD* = 2.17), and luteal phase ranged from day 3 post ovulation to 3 days before the onset of the next menstruation (*M*_day_ = 7.31, *SD* = 2.41, until onset of the following cycle). Cycle phases were additionally confirmed by salivary hormone levels and participants were excluded if the levels were not as expected for both hormonal values (see hormone analysis section).

Each session took place between 11 a.m. and 5 p.m., starting with participants answering a brief questionnaire about general habits and possible ongoing stressors, and followed by the scanning session. Saliva samples were collected before and after the scanning. Before the first session and during menses, women were screened for mental health problems and assessed their general intelligence through the Beck’s Depression and Anxiety Inventories (BDI2^69^; and BAI^70^) and the Ravens Advanced Progressive Matrices (APM), respectively.

### Cognitive paradigms

Stimulus presentation and acquisition of behavioral data were done with Presentation®^71^ (http://www.neurobs.com/) in a CRT monitor (1024 × 768 pixel resolution, 120 Hz refresh rate). Inhibitory control (IC) was assessed through a SST. There were two types of trial: “Go” and “Stop”. A green arrow (Go signal) appeared on the screen randomly oriented either to the left or to right (50% each). Participants were instructed to press the left or the right button depending on the arrow’s orientation. The arrow disappeared when the participant pressed the button or after 600 ms. Occasionally the green arrow changed to red (Stop signal), and participants needed to withhold their responses. The delay between the onset of the Go signal and the onset of the Stop signal or Stop signal delay (SSD) started at 250 ms and varied dynamically in a staircase tracking procedure to control inhibition probability^72^. Each trial’s SSD depended on individual performance in Stop trials: after a successfully inhibited Stop trial, the SSD in the next Stop trial increased by 50 ms, whereas when the participant was unable to stop the response, the SSD decreased by 50 ms in the next Stop trial. The entire SST consisted in four blocks of 120 trials each, being the first used as a training block and not included in the analysis. From the 120 trials 20 were null events (fixation cross) and 30 of them randomly change to red. The interstimuli interval varied randomly but equiprobably from 1250 to 1750 ms in steps of 125 ms. During these interstimuli intervals, a black cross as fixation point was presented.

Within the independent race model theoretical frame, Go and Stop processes are assumed to be stochastically independent and compete with each other, being the response inhibited if the stop process is faster than the go, and vice versa^1,2^. While duration of the Go process is directly observable through reaction time, the duration of the inhibitory process or Stop signal reaction time (SSRT) is covert and need to be computed from the observed effects of varying the SSD. With the staircase procedure, an optimal SSD in which motor actions were successfully inhibited in about half of the Stop trials could be estimated to calculate the SSRT. To determine it, we subtracted the mean SSD from the Go RT at the percentile corresponding to the proportion of unsuccessfully inhibited Stop trials as done before by previous studies^73,74^. This quantile approach is less susceptible to the individual deviations from even ratios of successful and unsuccessful inhibited trials and suggested as a more robust measure^75–77^.

Given that the SSRT has been shown to fluctuate across the menstrual cycle^29^, the SSRT from the menses session, when both estradiol and progesterone levels are low, was chosen as index of baseline IC. Based on previous studies contrasting short vs. long SSRT groups as measure of impulsiveness^9,43^, we used the baseline IC as a covariate of interest. Given the small sample of our study we kept the SSRT as a continuous variable to maintain the analyses power, instead of splitting the sample in two groups.

### MRI data acquisition and analyses

Whole-brain fMRI data were acquired on a Siemens Magnetom Trio Tim 3 Tesla scanner at the Christian Doppler Klinik (Salzburg, Austria). Structural images were obtained using a T1-weighted sagittal 3D MPRAGE sequence (TR = 2300 ms, TE = 2.91 ms, TI delay of 900 ms, FOV 256 mm, slice thickness = 1.00 mm, flip angle 9°, voxel size 1.0 × 1.0 × 1.0 mm, 160 sagittal slices). Functional scans were acquired descending interleaved with a T2*-weighted gradient echo planar (EPI) sequence sensitive to blood oxygen level dependent (BOLD) contrast. Three runs of approximately 4 min to 5 min each (depending on the speed of the participant, since the trials were response terminated) were acquired for the SST (TR = 2250 ms, TE = 30 ms, FOV 192 mm, matrix size 192 × 192, slice thickness = 3.0 mm, flip angle 70°, voxel size 3.0 × 3.0 × 3.0 mm, 36 transversal slices parallel to the AC-PC line).

For the pre-processing, the first 6 images of each scanning session were discarded and the remaining scans were despiked using 3d-despiking as implemented in AFNI (afni.nimh.nih.gov). Then, the images were realigned and unwarped using the Statistical Parametric Mapping package (SPM12, https://www.fil.ion.ucl.ac.uk/spm/software/spm12). For the identification and correction of non-physiological noise a biophysically-based model (Functional Image Artefact Correction Heuristic, FIACH^78^) was applied, the images were filtered and 6 regressors extracted. The filtered images were then subjected to the SPM12 standard pre-processing pipeline including slice timing, co-registration of functional to structural images, segmentation of structural images using CAT12 and normalization using the parameters obtained by CAT12 via the “pull” option (CAT, http://dbm.neuro.uni-jena.de/vbm/)^79^. Finally, data were resampled to isotropic 3 × 3 × 3 mm voxels and smoothed with a Gaussian kernel of 6 mm.

For statistical analysis we applied a two stage mixed effects model. In the subject-dependent fixed-effects first-level analysis, 3 different regressors were modelled separately to predict BOLD responses to the different types of trial outcome: Go hits, Stop inhibited and Stop non-inhibited. As done by Li *et al*.^40,80^ Go onsets were parametrically modulated by its corresponding reaction time (RT), and Stop inhibited and non-inhibited by its corresponding SSD. All regressors were obtained by convolving the duration of the event with the canonical hemodynamic response function implemented in SPM. The 6 realignment parameters and the 6 physiological noise parameters obtained from the FIACH procedure were entered as regressors of no interest, a high pass filter cut-off was set at 128 seconds and autocorrelation correction was performed using an AR(1) model^81^.

One statistical contrast was defined in this first level comparing the inhibited Stop responses to the non-inhibited. The contrast was scaled by dividing the contrast image by the amplitude of low frequency fluctuations (ALFF) map^82^ obtained from estimation residuals using the DPABI toolbox^83^ and a bandpass filter of 0.01–0.08 Hz. The scaled contrast images from each subject an session entered a One-sample t-test to assess the overall brain activation during the inhibited Stop responses compared to the non-inhibited Stops. Maximum global peak coordinates of activation above threshold were located in the left and right putamen ([−18, 8, −5], [21, 11, −5], respectively). Due to our focus on the basal ganglia (BG), we employed an explicit mask of bilateral caudate, putamen and pallidum created by the Wake Forest University (WFU) Pickatlas toolbox^84,85^ to examine the results of the One-sample t-test. Maximum global peak coordinates of activation above threshold were identical to the whole brain One-sample t-test ([−18, 8, −5], *k*_E_ = 151, *T* = 10.07, *p*_FWE_ = 0.000 and [21, 11, −5], *k*_E_ = 276, *T* = 10.79, *p*_FWE_ = 0.000). For the non-inhibited Stops responses compared to the inhibited Stops no voxel survived the pFWE < 0.05 at cluster level correction in these areas. Therefore, the left and right putamen were identified as the region of interest (ROI) for further analyses (Fig. 3a). Eigenvalues as measure of BOLD-response were extracted from a 6 mm sphere around these peaks restricted to the bilateral putamen using masks created by the WFU Pickatlas toolbox, and entered as dependent variables into a linear mixed model as further explained in the statistical analysis section.

In order to assess the connectivity of both left and right putamen, a psychophysiological interaction (PPI) analysis was performed^86,87^. To extract the raw time-course of activation for each subject, the parametric modulators were removed from the first-level model. The time-course was then obtained as the principle eigenvariate extracted from the peak coordinates described above. For each individual subject ROIs were allowed to shift from the peak coordinates to the nearest local maximum within a 6 mm radius, but confined to the left or right putamen as described above. This time-course was then multiplied with the hemodynamic response function (HRF) convolved regressors for inhibited responses vs. non-inhibited using the PPI toolbox. These PPI regressors represent increased connectivity during successfully inhibited Stop trials compared to non-inhibited Stop trials. The PPI time series, the original fMRI time series and the HRF convolved psychological variable were entered as regressors into a new first-level analysis without the parametric modulators. A contrast over the time-series regressor was defined for each participant at first level and entered into a whole brain One-sample t-test at second level, to identify brain areas where BOLD-response co-varies significantly with the activity of the putamen during inhibited Stop trials opposed to the non-inhibited. Finally, the contrasts from the first level were also entered (i) in a flexible-factorial design with within subject variable cycle phase (menses, pre-ovulatory and luteal), and (ii) in a flexible-factorial design adding the individual baseline IC as a covariate of interest, in order to evaluate (i) effect of cycle phase on putamen connectivity, and (ii) the interactive effect between cycle phase and individual level of IC on putamen connectivity.

For these analyses significance threshold was initially set at p < 0.001 uncorrected, and corrected to pFWE <0.05 at cluster level. Each peak-level Montreal Neurological Institute (MNI) coordinate was labelled using the Automated Anatomical Labeling toolbox (AAL^85^). In order to attribute the observed effects to either estradiol or progesterone, mean eigenvalues were extracted from significant clusters and entered into a linear-mixed effects (LME) analysis as described in the statistical analysis section.

### Hormone analysis

In order to assess estradiol and progesterone levels two saliva samples 2 ml each were collected via the passive drool method before and after every session and stored at −20 °C until hormone assessment. Solid particles were removed by centrifugation (3000 rpm for 15 min, then 3000 rpm for 10 min) and pooled in order to ensure reliability of hormone assessment over the course of the scanning session. Estradiol was quantified using Salimetrics High Sensitivity salivary estradiol assays and progesterone levels were quantified using DeMediTec Diagnostics ELISA kits. Samples of one subject were always analyzed on the same plate, but multiple plates were needed for the study. The average of duplicate values was used for statistical analyses. One of the estradiol samples was excluded due to visible blood contamination. Hormone values were used (a) as exclusion criteria for participants with a mismatch between the actual and expected hormonal profile, and (b) as covariates in the statistical analyses to explore whether the cycle changes observed were attributable to estradiol or progesterone. Participants were excluded if their progesterone was not highest during the luteal phase and their estradiol levels were highest during menses (3 participants).

### Statistical analysis

To ensure the success of the tracking algorithm procedure in the SST, that leads to approximately half of the Stop trials presented unsuccessfully inhibited, we excluded those participants (n = 3) whose hit Stop percentage was in the range of two standard deviations above or below the mean 53.06 ± (2*9.29). The data from the remaining 36 participants was analysed in R 3.2.2. SSRT was not correlated to any of the initial neuropsychological measures from the BDI2^69^; BAI^70^, and APM test (all |*cor*| < 0.25, all |*t*| < 1.50, all *p* > 0.05). Session did not affect behaviour or neuroimaging parameters (all |*b*| < 0.15, all SE_b_ < 0.10, all |*t*| < 1.90, all *p* > 0.05), and was therefore not considered further.

In order to analyze the behavioral data, the bilateral putamen BOLD-response and connectivity, and the effects of hormonal levels, statistical analyses were carried out assessing the possible effect of the factors in each dependent variable through LME models. Each behavioral measure, and the principal eigenvariates from the bilateral putamen for activation and connectivity were the dependent variable, the participant number (PNr) was included as random factor, and cycle phase and the baseline IC, as fixed variables (e.g. BOLDPut ~ 1|PNr + cycle*SSRT menses). All models were rerun after excluding menses to explore the differences between the pre-ovulatory phase and the luteal phase. To further analyse whether the cycle effects were attributable to estradiol or progesterone, those models that showed a cycle phase effect were rerun with the fixed variables that showed any significant effect, replacing cycle phase by estradiol and progesterone values, respectively (e.g. BOLDPut ~ 1|PNr + hormone*SSRT menses). Finally, a model was run with the SSRT as dependent variable, and the bilateral putamen BOLD-response and connectivity as fixed variables, in order to explore which of them could predict the behavioral performance (e.g. SSRT ~ 1|PNr + BOLDPut). The baseline IC and hormonal values were included as continuous variables, while cycle phase was factorized. In all models, both the dependent and continuous independent variables were z-standardized using the scale function. Therefore, the coefficients b of fixed effects represent a standardized effect size based on standard deviations, similar to Cohen’s d. Data and scripts are openly available at http://webapps.ccns.sbg.ac.at/OpenData/. MR-images are available upon request from the first author.
